# Supplementary material for: Revealing the intrinsic nature of the mid-gap defects in amorphous Ge2Sb2Te5
Source: Nat Commun. 2019 Jul 11;10:3065. doi: 10.1038/s41467-019-10980-w (PMC6624207; doi:10.1038/s41467-019-10980-w)
Supplement: Supplementary file 1 — Supplementary Information [file 41467_2019_10980_MOESM1_ESM.pdf]

Revealing the intrinsic nature of the mid-gap defects in amorphous $Ge_2Sb_2Te_5$

Konstantinou et al.

Supplementary Information

Supplementary Notes

Supplementary Note 1. The concentration of the in-gap electronic states in amorphous $Ge_2Sb_2Te_5$ (225GST) phase-change memory material is relatively low, which makes these defects difficult to be identified with computational modelling. Hence, the study of mid-gap defect states in amorphous 225GST requires calculations for many glass models in order to credibly predict the mid-gap defect configurations, and to obtain a statistically significant understanding.

The most popular method of generating amorphous structures is based on melting crystalline or random-structure samples in simulation cells with 3D periodic boundary conditions, and the subsequent cooling of the melt at different rates to low temperatures to obtain glassy structures[1]. This method is based on the assumption that all liquids can be frozen into an amorphous state by sufficiently fast cooling. The melt-and-quench method can be applied either by using first-principles molecular-dynamics simulations or by combining classical molecular-dynamics simulations, using interatomic force fields, with a subsequent possible geometry optimization of the obtained structures using density-functional theory (DFT). It is worth emphasizing that, when reliable force fields are available, the second approach is more efficient for producing many models of an amorphous structure.

Ab initio DFT-based molecular-dynamics simulations are computationally very demanding and prohibitive to be employed in order to generate many amorphous models, and in simulation studies of amorphous 225GST, typically only one to three small periodic supercell models have been generated[2-4]. Classical molecular-dynamics simulations of amorphous 225GST were hampered till recently by the lack of suitable (empirical) force fields to describe the complex bonding interactions present in the material.

In this study, the recently-developed, machine-learned, DFT-accurate $O(N)$ Gaussian approximation potential (GAP) for the phase-change memory material, Ge-Sb-Te, from our group[5] has been used to generate a database of independent amorphous 225GST structures via classical molecular-dynamics simulations. Hybrid-DFT calculations were used to optimise the geometry of all the glass models and to calculate their electronic structures. The calculations performed in this work produce sufficient statistics and allow us to identify the structural motifs responsible for the mid-gap states in the band gap of the amorphous material under study. The results from the calculations for a 900-atom glass structure complement the observations, increase the overall statistical accuracy of the study, and strengthen the conclusions for the atomistic nature of the mid-gap defect electronic states expected to be present in a real sample of amorphous 225GST.

Supplementary Note 2. The first, second and third nearest-neighbour distances, corresponding to the first three peaks in the radial distribution function, shown in **Supplementary Figure 1**, are 2.89 Å, 4.09 Å and 6.23 Å, respectively, for the 225GST glass models generated by using the Gaussian approximation potential (GAP), and 2.89 Å, 4.15 Å and 6.29 Å, respectively, for the amorphous 225GST model generated by *ab initio* density-functional theory (DFT) molecular-dynamics (MD) simulation[4]. This comparison highlights the very good quantitative agreement between the local atomic structure of the GAP-MD glasses and the DFT-MD amorphous model. Beyond these peaks, it can be observed that the agreement is less good between the glass models, since the GAP-MD samples do not quite capture correctly the fourth and fifth peaks in the *g(r)* of the DFT structure. However, this discrepancy is not too significant, since the local atomic structure of the glass is mainly described within the first and second coordination shells, which are also important with respect to our observations for the localization of the mid-gap defect states within the amorphous network.

The first peak in the total X-ray structure factor, shown in **Supplementary Figure 2**, is located at 2.07 $Å^{-1}$ in the experimental *S(q)* distribution[6], at 2.11 $Å^{-1}$ in the *S(q)* from the amorphous 225GST model generated by *ab initio* DFT molecular-dynamics simulation[4], and at 2.14 $Å^{-1}$ in the *S(q)* calculated from the GAP-MD 225GST glasses generated in this work. In addition, the second and third peaks are located at 3.37 $Å^{-1}$ and 5.16 $Å^{-1}$, 3.28 $Å^{-1}$ and 4.97 $Å^{-1}$, 3.27 $Å^{-1}$ and 4.94 $Å^{-1}$, in the same S(q) distributions, respectively. Overall, it can be observed that the quantitative comparison between the peak positions in the relative *S(q)* distributions indicates very good agreement between the experimental data and the amorphous 225GST models generated in our current study, with respect to the local atomic structure of the GAP-MD glasses, highlighting that the glass models describe with considerable success the short-range order and they are indeed representative of amorphous 225GST.

Supplementary Note 3. The smooth overlap of atomic potentials (SOAP) structural descriptor[7] was applied to describe the local environment of each atomic species in all the glass models generated in this work. Each descriptor is a vector existing in a high-dimensional space, while a measurement of the similarity between two local environments is given by the dot-product of these vectors. The SOAP descriptors are normalized such that the dot-product of any two of them lies between 0, corresponding to no similarity whatsoever, and 1, corresponding to two identical local environments. This approach has been used previously for the visualization of structural datasets of several materials and molecules[8], while recently

it was applied in order to investigate the similarity between amorphous titania and its crystalline polymorphs[9].

Multidimensional scaling (MDS)[10] is a non-linear dimensionality-reduction technique that can be used to visualize the SOAP data in a two-dimensional space. In this way, relative descriptor-distances are maintained, and an accurate two-dimensional representation of the many-dimensional data is obtained. Hence, with appropriate algorithms, the high-dimensional SOAP data can be embedded into two-dimensional maps in which they can be interpreted accordingly. As the high-dimensional pairwise distances are approximately preserved in the two-dimensional representation, such maps can be used to search for structurally distinct local environments that correspond to coordination defects, like those for instance related to Ge local environments which were identified to be responsible for the mid-gap electronic states in the band gap of amorphous 225GST.

Supplementary Note 4. The coordination numbers of the three atomic species in the simulated amorphous 225GST structures, as obtained from an Electron-Localization Function (ELF) analysis[11,12], are shown in **Supplementary Figure 34**. It can be observed that 3- or 4-fold coordination is the favourable arrangement for Ge local environments, while 3-fold coordinated environments are the most probable for Sb and Te atoms. The ELF analysis results in slightly smaller average coordination numbers for the three atomic species in the glass structures, while the geometrical bond-distance cut-off approach shows slightly over-structured local environments for the glass models. However, the differences are not very significant, since both approaches highlight, for instance, the large range of Ge local environments inside the glass structure.

The amount of 5- and 6-coordinated Ge atoms was found to be 18% with the geometrical bond-distance cut-off approach, while with the ELF analysis, 11% of the Ge atoms were found to have 5 or 6 atoms in their nearest surroundings. However, it is worth highlighting that, even though the geometrical bond-distance cut-off approach somewhat over-estimates the amount of 5-coordinated Ge atoms, the ELF analysis showed that this Ge local geometry is evidently present in the structural motif responsible for the localization of the mid-gap defects within the amorphous network of the glass samples, which verifies the crucial role of the 5-coordinated Ge atoms in the local atomic structure of the mid-gap defects.

It is worth noting that an optimal geometric bond-distance cut-off, that minimizes the number of errors, can be deduced from an electronic-structure calculation in several different ways[13,14]. A value for this cut-off between 3.0 and 3.2 Å is typically obtained for amorphous 225GST, which highlights that the radial cut-off distance of 3.2 Å used in our work for the structural analysis corresponds to a reasonable choice for describing the chemical bonds in

225GST in a correct manner. Overall, the geometrical bond-distance cut-off approach, combined with the SOAP structural descriptors for the local environments of the atomic species in the glass samples, correspond to a very good description of the local atomic structure of the amorphous models.

Supplementary Note 5. There are several studies in the literature about the structure of amorphous 225GST reporting the effects of different DFT-schemes, with respect to the exchange-correlation functional[15,16] and the pseudopotential[17] used within the generalized-gradient approximation (GGA), on the quality of the generated models. It has been observed that GGA calculations with the PBE functional result in longer Ge–Te and Sb–Te bond lengths compared to the experimental local atomic structures in glassy 225GST, revealing, small, but significant discrepancies[18]. A geometry optimization with hybrid-DFT calculations decreased the estimated Ge–Te and Sb–Te interatomic distances in the simulated amorphous 225GST, leading to better agreement with the experimental structure compared to the GGA geometry relaxations[18]. This observation validates our choice of using a nonlocal functional for the geometry optimization of the generated GAP-MD glass models, which already had a near-GGA accuracy. Moreover, the inclusion of a portion of the Hartree-Fock exchange to the PBE approximation is imperative in order to be able to improve significantly the estimation of the band gap, and hence establish a better description of the electronic structure, which is crucial for the investigation of the mid-gap electronic states in amorphous 225GST. Hybrid-functional DFT calculations are essential to identify the localized defect states in the band gap of glassy 225GST, which then can be ascribed to specific structural features within the amorphous network.

Supplementary Note 6. In order to explore the similarity of the generated amorphous models, the global SOAP descriptor[7] can be used for the structure of each glass sample as a whole, and the results are shown in the configurational map presented in **Supplementary Figure 35**, in which twenty liquid models and an ideal crystalline configuration were added as landmarks, for comparison purposes. The SOAP descriptor takes all structural aspects of the three phases into account, providing a distance measure for similarity between liquid, glass and crystal, yielding an $N \times N$ matrix, where N is the number of structures investigated. Metric MDS algorithms were used to visualize these data in a two-dimensional space[10]. In the SOAP-based similarity map, each point corresponds to a different structural 225GST model, while the relative distances between the individual points are correlated with the structural similarity of

the different samples. It can be observed that regions corresponding to the liquid, amorphous and crystalline structures are clearly separated. However, glass models with in-gap states (highlighted with green points) cannot be easily distinguished from their amorphous counterparts without any defect states in their band gap (highlighted with red points).

Nevertheless, it was observed that 5-coordinated Ge atoms correspond to a defective local environment responsible for the localisation of the mid-gap states inside the glass. For this reason, the calculated Ge local environments in the glass models with defect gap-states were compared to those in the glass samples with a clean band gap. The Ge coordination numbers were computed by using the same geometrical bond-distance cut-off as before (3.2 Å) and the results are shown in **Supplementary Figure 36**. One can observe that, in the glass models with mid-gap electronic states, the amount of 5- and 6-coordinated Ge atoms is larger than that in the glasses without defect states, highlighting a structural difference between the two types of samples. In addition, the number of 3-coordinated Ge atoms is also larger in the glasses with mid-gap states, whereas the amount of 4-coordinated Ge atoms is larger in the models without in-gap states, indicating, overall, the presence of more defective Ge local environments within the amorphous network of the samples with in-gap electronic states in their band gap.

Overall, the SOAP analysis indicates that the atomic structures of the different amorphous models generated in this work are quite similar, with respect to the first- and second-nearest neighbours. However, based on the localization of the mid-gap states within the amorphous network and the analysis for the Ge local environments in the glass models with and without in-gap states, some relatively subtle structural differences can be observed, which provide some sense of the structural dissimilarity with respect to the atomic geometry between the two types of glass samples.

Supplementary Note 7. A SOAP map in which the distribution of points from the different amorphous models is compared to crystalline-like environments is shown in **Supplementary Figure 38**. Local environments from crystalline configurations, as well as those from the fragments in which the identified mid-gap states are localized within the amorphous network of the modelled systems, are found to be towards the edges of each species cluster, and, in particular, in regions corresponding to over-coordinated atoms.

Supplementary Note 8. The stress tensor for each atomic species in the 315-atom 225GST model was calculated by using the GAP framework[19,20]. The stress tensor (σ) corresponds to the derivative of the total energy with respect to scaling of the cell encoded in the strain (ϵ):

$$\sigma_{\alpha\beta} = -\frac{1}{\Omega}\frac{\partial E}{\partial \epsilon_{\alpha\beta}} \quad (1)$$

in which α and β are cartesian components, and Ω is the volume. The GAP model provides a local decomposition of the total energy, resulting from the local atomic environments, indexed here by i:

$$E = \sum_i E_i \quad (2)$$

Hence, in this case, by taking the derivatives with respect to the cell vectors, and scaling with the total volume, on both sides of the equation above, the total stress can be written as a sum of local atomic virial stresses:

$$\sigma = \sum_i -\frac{1}{\Omega}\frac{\partial E_i}{\partial \epsilon_{\alpha\beta}} = \sum_i \sigma_i \quad (3)$$

The trace of the stress tensor will give then a local atomic pressure for each atom in the modelled glass system, which corresponds to the property that was shown in Figure 5 of the paper:

$$p_i = \frac{1}{3} Tr[\sigma_i] \quad (4)$$

It is noted that the total value of the stress in the glass structure is comparable between DFT and GAP calculations, since the GAP potential was trained using stress values from the DFT data that correspond to the reference training set[5].

Supplementary Note 9. The density of the glass models was fixed to the experimentally reported value for amorphous 225GST[21], since, from the simulations presented in this work, we did not aim to predict the density of the glass. Fixing the computational cell volume to match the target-glass density throughout the melt-and-quench molecular-dynamics simulations is a robust approach that can be employed to generate glass models with good representative atomic structures, which is important in finding the structural motifs within the amorphous network that are hosting the mid-gap defect electronic states of the glass. However, the fixed-volume approach will result in a glass structure with residual stress in the cell. In the thirty 225GST amorphous models generated in this work, the total residual stress ranged between 1.3 and 1.7 GPa in the glass structures after geometry optimizations with the hybrid-DFT calculations. We believe, though, that this does not affect the quality of the

calculations, since the effect of the cell relaxation is expected to be small on the volume of the simulated systems, as reported before from similar calculations in amorphous materials[22].

Supplementary Note 10. For the simulations presented in this work, each glass model was generated from scratch. We did not quench from the same liquid structure, for example (which is a virtually independent approach), to obtain amorphous structures, nor did we just select snapshots from a trajectory at a specific temperature (300K) to accumulate glassy atomic environments. In this study, for each glass sample, the atoms, initially, were placed in a pseudo-random arrangement (with a different seed in each case) inside a cubic simulation box. Every system was melted at a high temperature (3000K) for a sufficiently long time (30ps) to ensure that any memory effect of the initial pseudo-random guess of the atomic positions is lost before the system was equilibrated to the liquid temperature and then subsequently quenched. For the melting (3000K) and liquid (1200K) temperatures, different seeds were also used for the velocity distributions before starting the MD runs for each model. Finally, it is important to realize that each configuration was subjected to independent NVT runs during the molecular-dynamics simulations of the glass samples.

Supplementary Note 11. The study performed in this work involves a two-step approach for generating GAP-MD amorphous models and then calculating their electronic structure with hybrid-DFT geometry optimizations. PBE0 was chosen as the hybrid functional for our calculations[23], which is implemented efficiently within the CP2K code[24] using the truncated Coulomb operator together with the GTH pseudopotentials[25], while also it offers more consistency with the GAP glass models over other hybrid functionals. In general, performing hybrid-DFT calculations with the CP2K code is very efficient, since the computational cost of non-local functional calculations can be reduced using the auxiliary density-matrix method (ADMM)[26]. With this approach, the density is mapped onto a much sparser Gaussian basis set containing less diffuse and fewer primitive Gaussian functions than the one employed in the rest of the calculation. This allows the Hartree-Fock exchange terms, whose computational expense scales as the fourth power of the number of basis functions, to be calculated on a much smaller basis set for the rest of the calculation, which substantially reduces the computational time[27]. Overall, we would like to note that our approach followed in the calculations presented in this work has a very good combination of accuracy, efficiency and transferability with respect to the objective of our study in identifying the localized mid-gap electronic states present in the band gap of glassy 225GST and revealing the local atomic structure that hosts these defects within the amorphous network.

Supplementary Figures

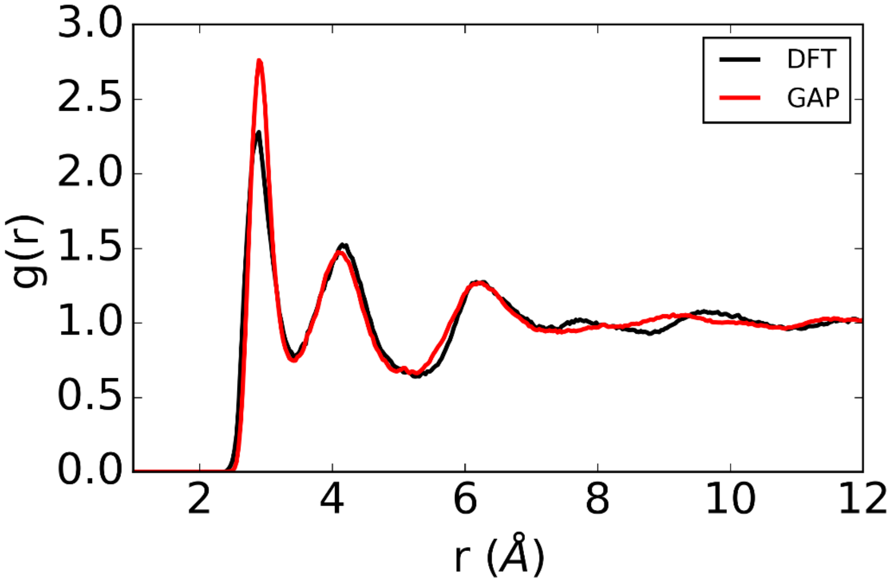

Supplementary Figure 1 | Comparison of radial distribution functions. Total radial distribution function calculated, on average, from the thirty amorphous 225GST models generated in this work using the GAP potential. Comparison is made with a *g(r)* calculated from a structural model of amorphous 225GST, with the same system size, generated by *ab initio* DFT molecular-dynamics simulation in a previous work of our group[4]. No significant differences can be observed between the amorphous structures generated with DFT and the GAP potential, indicating the high level of accuracy of the interatomic potential with respect to the short-range order of amorphous 225GST.

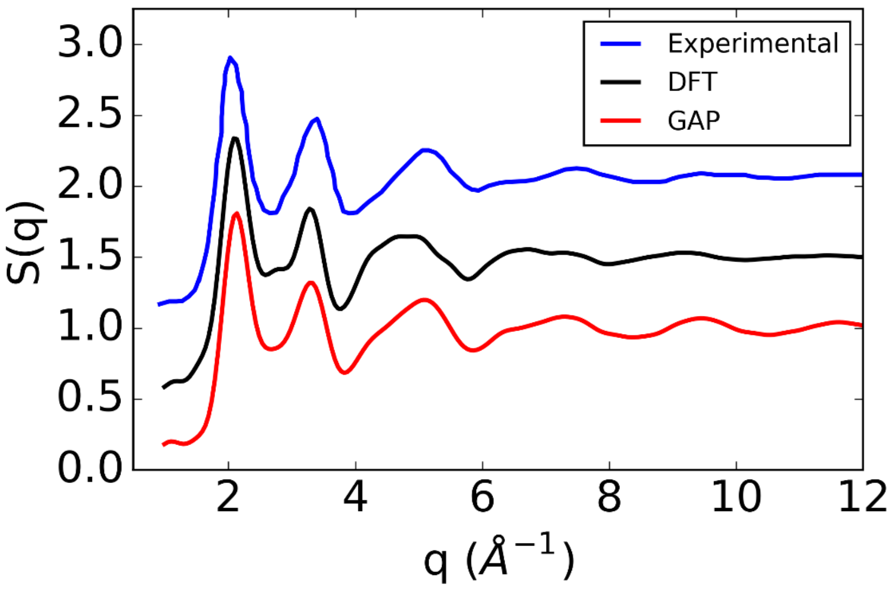

Supplementary Figure 2 | Comparison of structure factors. Calculated total X-ray structure factor of the simulated amorphous 225GST structures. Comparison is made with an experimental *S(q)* from X-ray diffraction data, taken from the literature[6], and with the total *S(q)* of an amorphous 225GST model of the same size as the glass models in this work, generated by *ab initio* DFT molecular-dynamics simulation in a previous work of our group[4]. Simulated and experimental results are in very good agreement, and this comparison highlights the quality of the amorphous models generated in this work. It is noted that the experimental structure factor and that calculated for the DFT glass model have been shifted vertically by 1.0 and 0.5 units, respectively, for clarity.

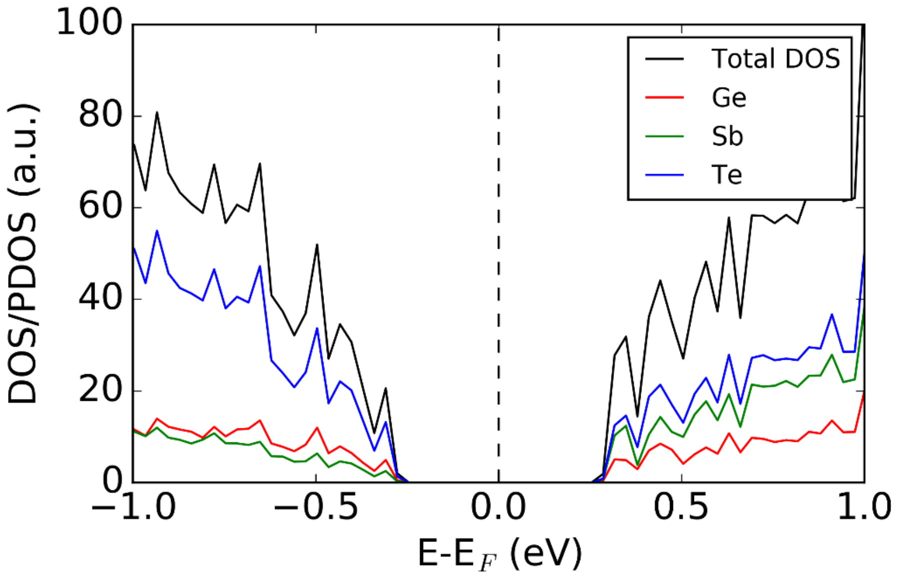

Supplementary Figure 3 | DOS/PDOS glass model #3. Total and partial electronic densities of states near the top of the valence band and the bottom of the conduction band of the 225GST glass model #3 from Supplementary Table 1, which does not have a mid-gap defect state in the band gap. The calculated HOMO-LUMO band gap is 0.64 eV. The Fermi level lies approximately at the middle of the energy band gap. The bottom of the conduction band is due to Te and Sb states, and the top of the valence band is dominated by Te-atom states.

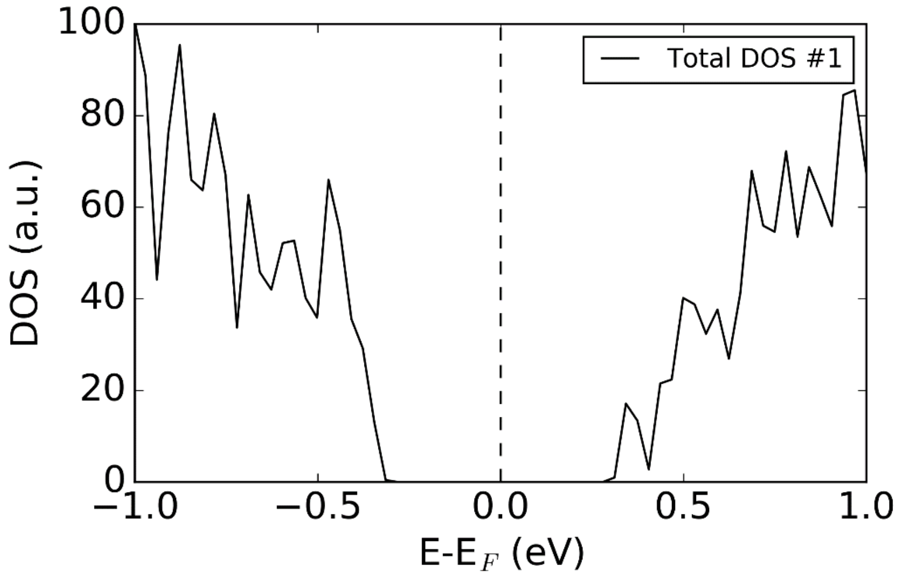

Supplementary Figure 4 | DOS glass model #1. Total electronic density of states near the top of the valence band and the bottom of the conduction band for the amorphous model #1. The calculated HOMO-LUMO band gap is 0.72 eV and this glass structure does not have a mid-gap defect state in the band gap. The Fermi level lies approximately at the middle of the energy band gap.

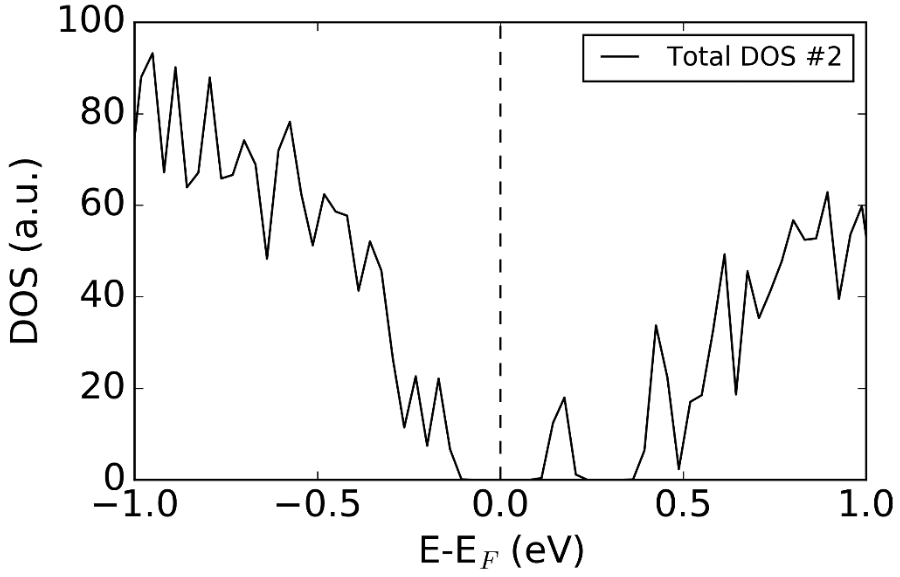

Supplementary Figure 5 | DOS glass model #2. Total electronic density of states near the top of the valence band and the bottom of the conduction band for the amorphous model #2. This glass structure has a mid-gap defect state located at 0.27 eV below the bottom of the conduction band, while the band gap of the model is 0.59 eV. The Fermi level lies deep in the band gap of the glass model, at a position between the mid-gap electronic state and the valence-band maximum.

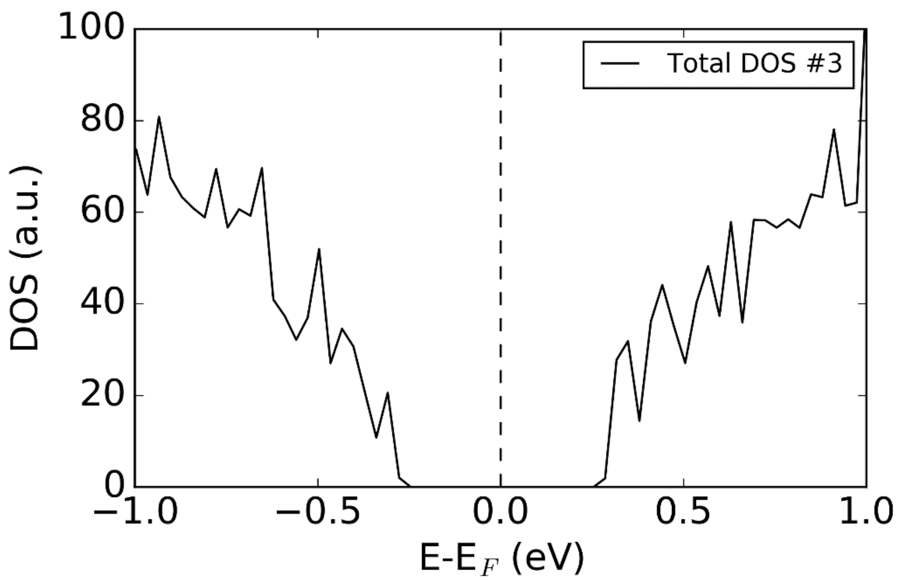

Supplementary Figure 6 | DOS glass model #3. Total electronic density of states near the top of the valence band and the bottom of the conduction band for the amorphous model #3. The calculated HOMO-LUMO band gap is 0.64 eV and this glass structure does not have a mid-gap defect state in the band gap. The Fermi level lies approximately at the middle of the energy band gap.

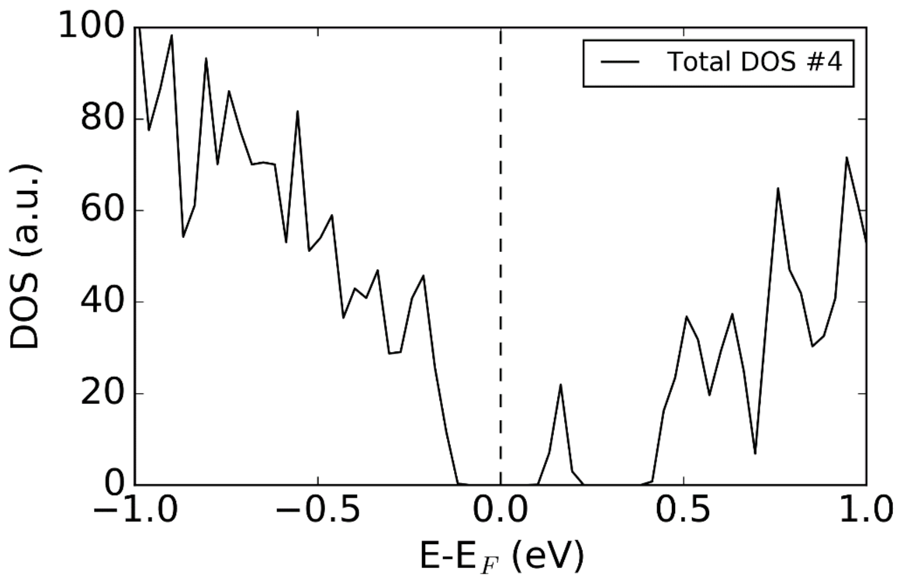

Supplementary Figure 7 | DOS glass model #4. Total electronic density of states near the top of the valence band and the bottom of the conduction band for the amorphous model #4. This glass structure has a mid-gap defect state located at 0.3 eV below the bottom of the conduction band, while the band gap of the model is 0.63 eV. The Fermi level lies deep in the band gap of the glass model, at a position between the mid-gap electronic state and the valence-band maximum.

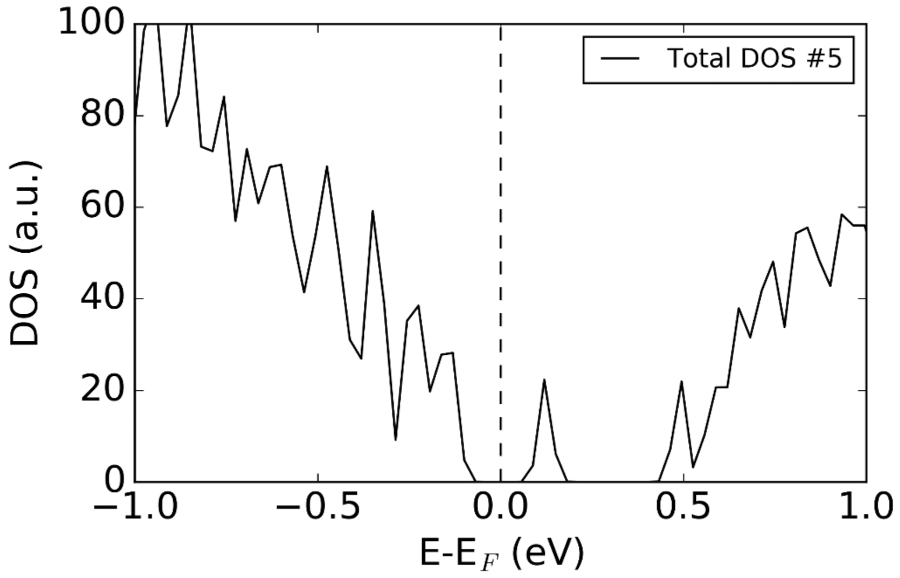

Supplementary Figure 8 | DOS glass model #5. Total electronic density of states near the top of the valence band and the bottom of the conduction band for the amorphous model #5. This glass structure has a mid-gap defect state located at 0.37 eV below the bottom of the conduction band, while the band gap of the model is 0.62 eV. The Fermi level lies deep in the band gap of the glass model, at a position between the mid-gap electronic state and the valence-band maximum.

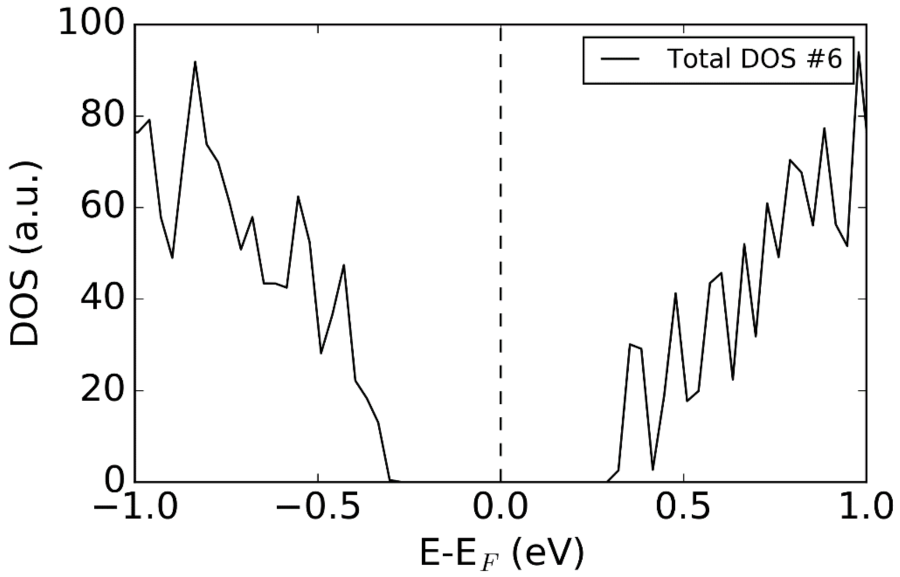

Supplementary Figure 9 | DOS glass model #6. Total electronic density of states near the top of the valence band and the bottom of the conduction band for the amorphous model #6. The calculated HOMO-LUMO band gap is 0.71 eV and this glass structure does not have a mid-gap defect state in the band gap. The Fermi level lies approximately at the middle of the energy band gap.

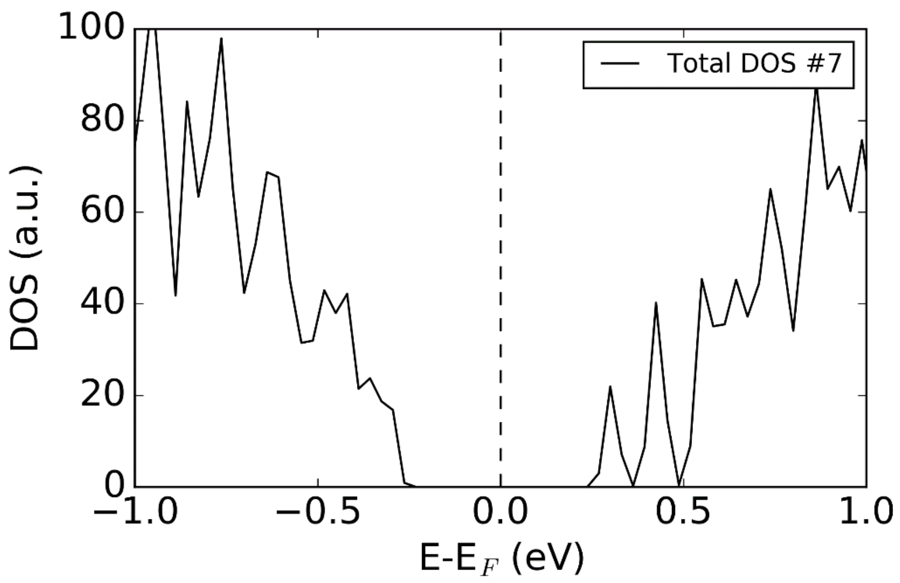

Supplementary Figure 10 | DOS glass model #7. Total electronic density of states near the top of the valence band and the bottom of the conduction band for the amorphous model #7. The calculated HOMO-LUMO band gap is 0.61 eV and this glass structure does not have a mid-gap defect state in the band gap. The Fermi level lies approximately at the middle of the energy band gap.

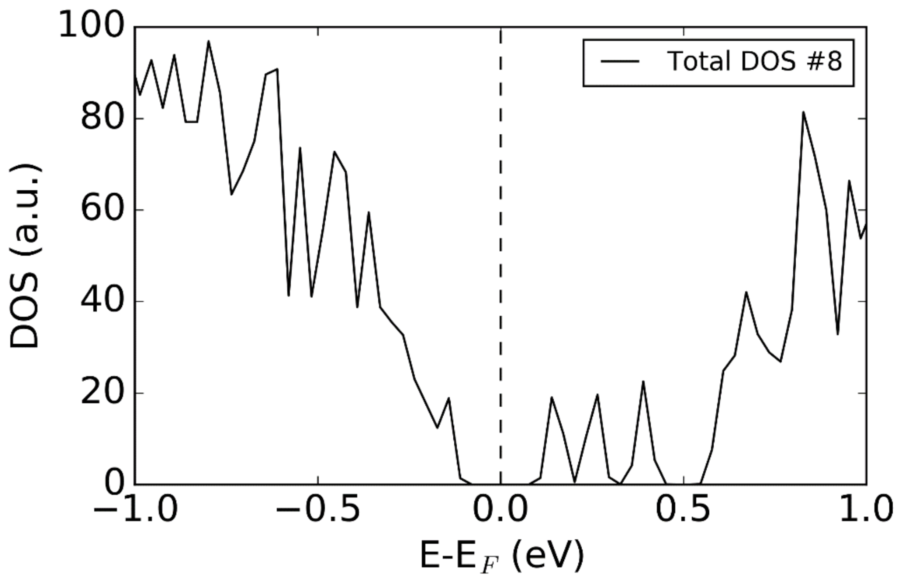

Supplementary Figure 11 | DOS glass model #8. Total electronic density of states near the top of the valence band and the bottom of the conduction band for the amorphous model #8. This glass structure has a mid-gap defect state located at 0.45 eV below the bottom of the conduction band, while the band gap of the model is 0.76 eV. Two more defect states, at 0.21 eV and 0.35 eV, also exist in the band gap of the glass structure. The Fermi level lies deep in the band gap of the glass model, at a position between the lowest mid-gap electronic state and the valence-band maximum.

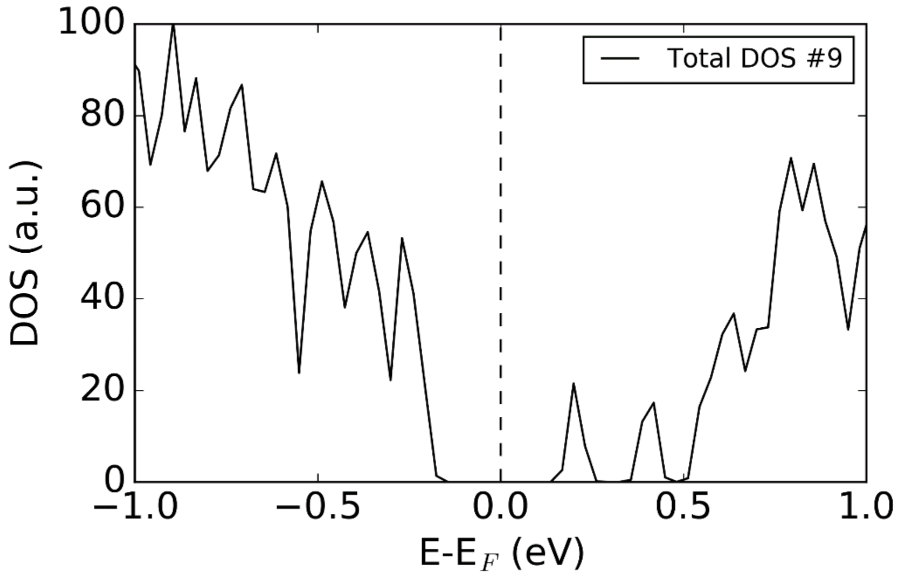

Supplementary Figure 12 | DOS glass model #9. Total electronic density of states near the top of the valence band and the bottom of the conduction band for the amorphous model #9. This glass structure has a mid-gap defect state located at 0.35 eV below the bottom of the conduction band, while the band gap of the model is 0.78 eV. A second, shallow, defect state is located at 0.15 eV in the band gap of the glass structure. The Fermi level lies deep in the band gap of the glass model, at a position between the mid-gap electronic state and the valence-band maximum.

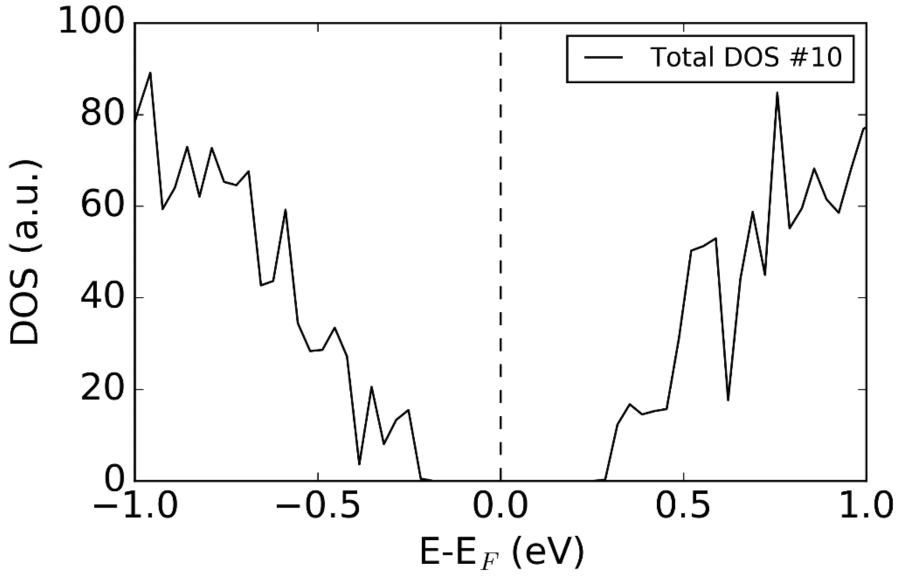

Supplementary Figure 13 | DOS glass model #10. Total electronic density of states near the top of the valence band and the bottom of the conduction band for the amorphous model #10. The calculated HOMO-LUMO band gap is 0.61 eV and this glass structure does not have a mid-gap defect state in the band gap. The Fermi level lies approximately at the middle of the energy band gap.

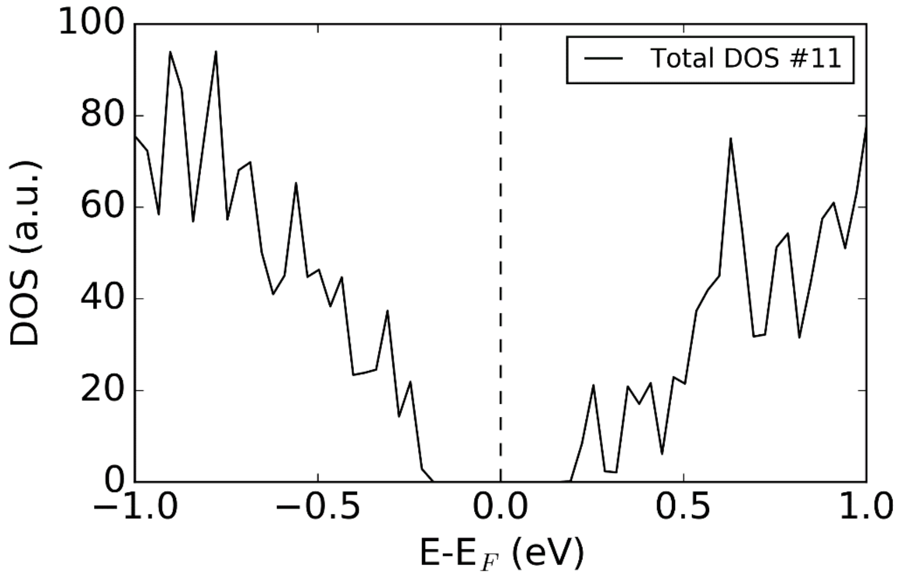

Supplementary Figure 14 | DOS glass model #11. Total electronic density of states near the top of the valence band and the bottom of the conduction band for the amorphous model #11. The calculated HOMO-LUMO band gap is 0.55 eV and this glass structure does not have a mid-gap defect state in the band gap. The Fermi level lies approximately at the middle of the energy band gap.

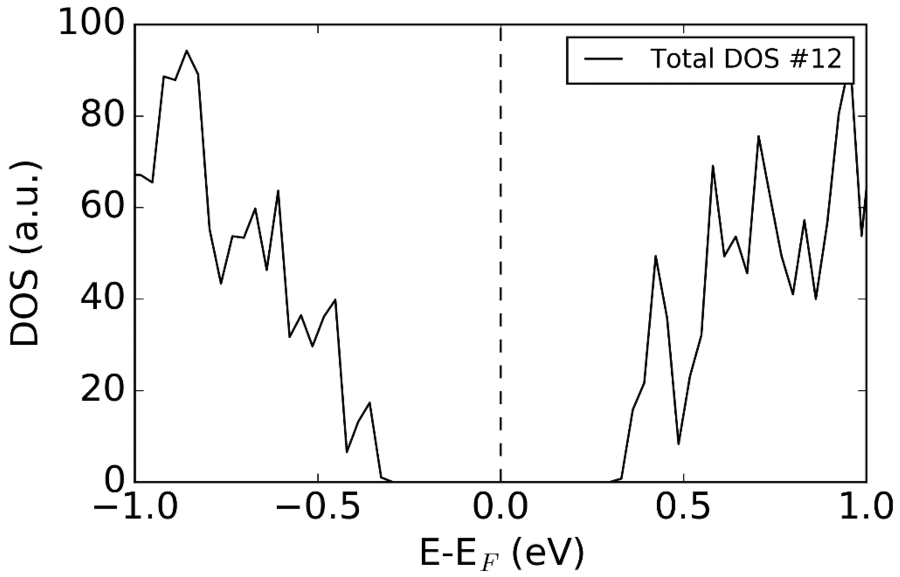

Supplementary Figure 15 | DOS glass model #12. Total electronic density of states near the top of the valence band and the bottom of the conduction band for the amorphous model #12. The calculated HOMO-LUMO band gap is 0.75 eV and this glass structure does not have a mid-gap defect state in the band gap. The Fermi level lies approximately at the middle of the energy band gap.

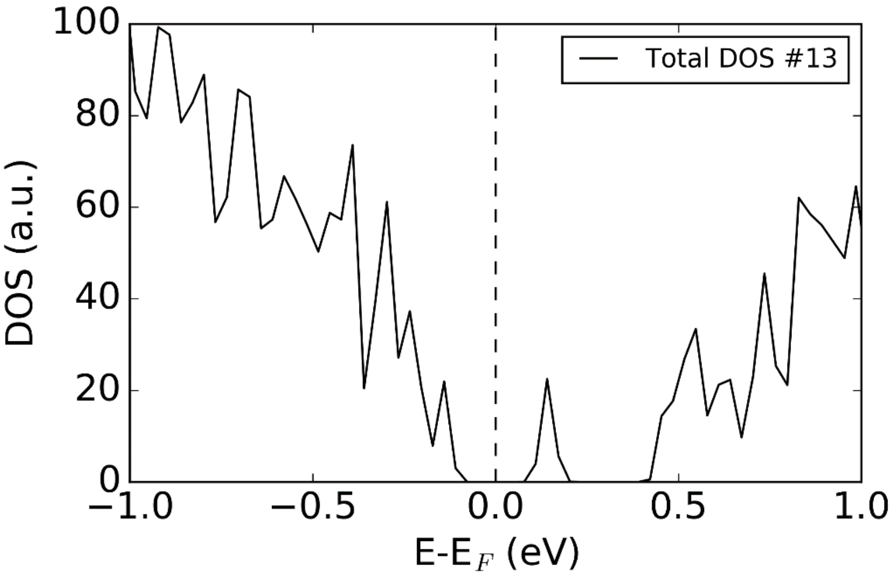

Supplementary Figure 16 | DOS glass model #13. Total electronic density of states near the top of the valence band and the bottom of the conduction band for the amorphous model #13. This glass structure has a mid-gap defect state located at 0.33 eV below the bottom of the conduction band, while the band gap of the model is 0.62 eV. The Fermi level lies deep in the band gap of the glass model, at a position between the mid-gap electronic state and the valence-band maximum.

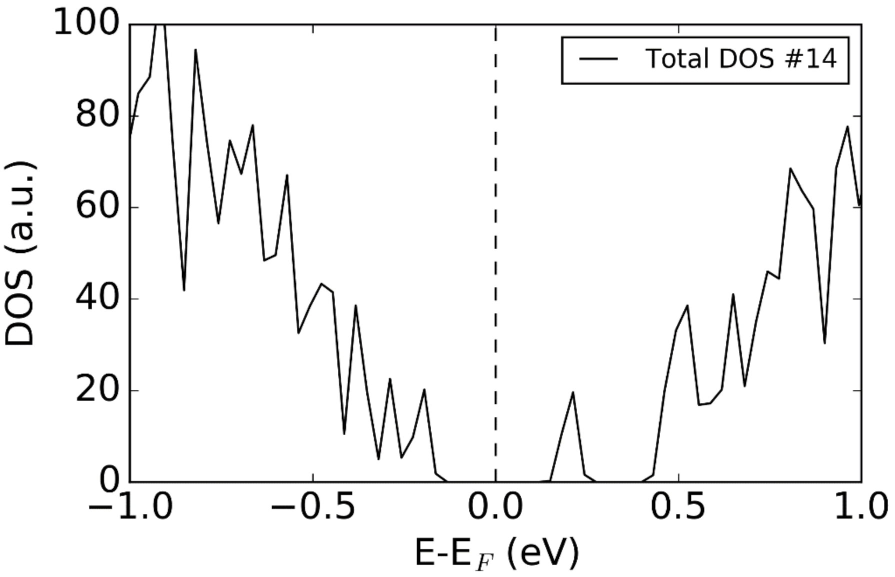

Supplementary Figure 17 | DOS glass model #14. Total electronic density of states near the top of the valence band and the bottom of the conduction band for the amorphous model #14. This glass structure has a mid-gap defect state located at 0.27 eV below the bottom of the conduction band, while the band gap of the model is 0.68 eV. The Fermi level lies deep in the band gap of the glass model, at a position between the mid-gap electronic state and the valence-band maximum.

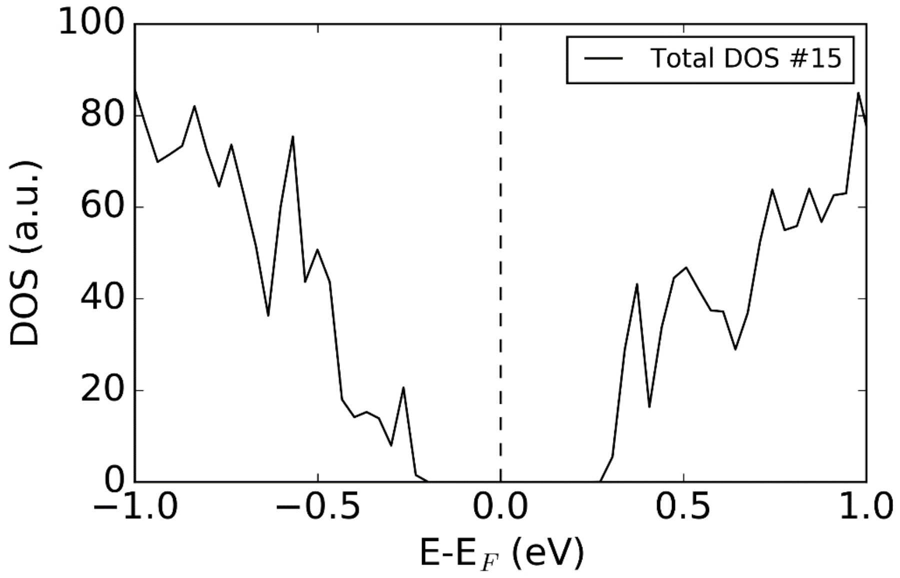

Supplementary Figure 18 | DOS glass model #15. Total electronic density of states near the top of the valence band and the bottom of the conduction band for the amorphous model #15. The calculated HOMO-LUMO band gap is 0.61 eV and this glass structure does not have a mid-gap defect state in the band gap. The Fermi level lies approximately at the middle of the energy band gap.

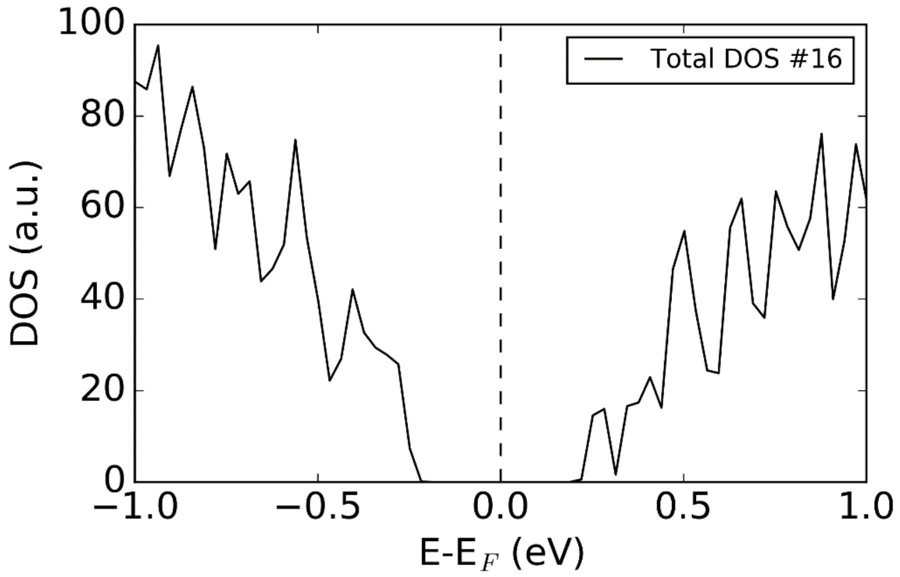

Supplementary Figure 19 | DOS glass model #16. Total electronic density of states near the top of the valence band and the bottom of the conduction band for the amorphous model #16. The calculated HOMO-LUMO band gap is 0.55 eV and this glass structure does not have a mid-gap defect state in the band gap. The Fermi level lies approximately at the middle of the energy band gap.

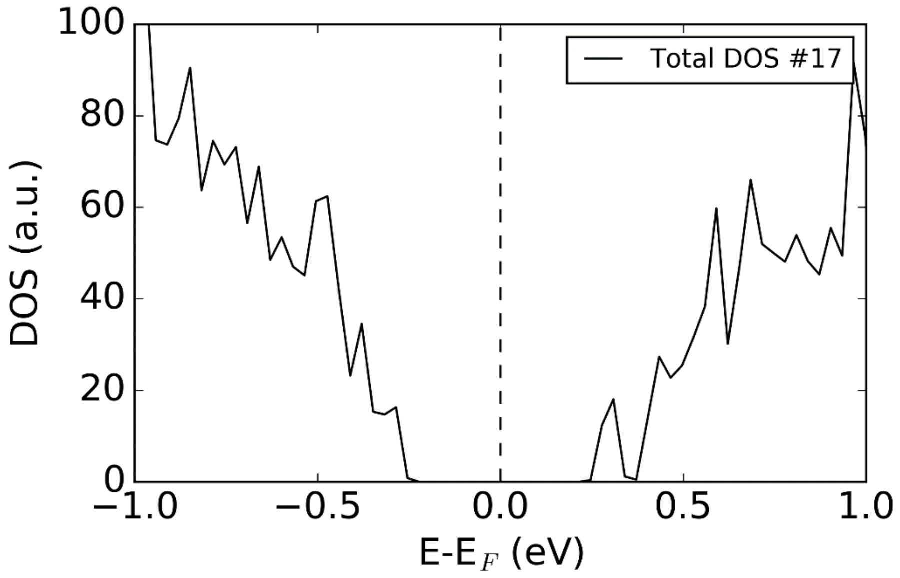

Supplementary Figure 20 | DOS glass model #17. Total electronic density of states near the top of the valence band and the bottom of the conduction band for the amorphous model #17. The calculated HOMO-LUMO band gap is 0.60 eV and this glass structure does not have a mid-gap defect state in the band gap. The Fermi level lies approximately at the middle of the energy band gap.

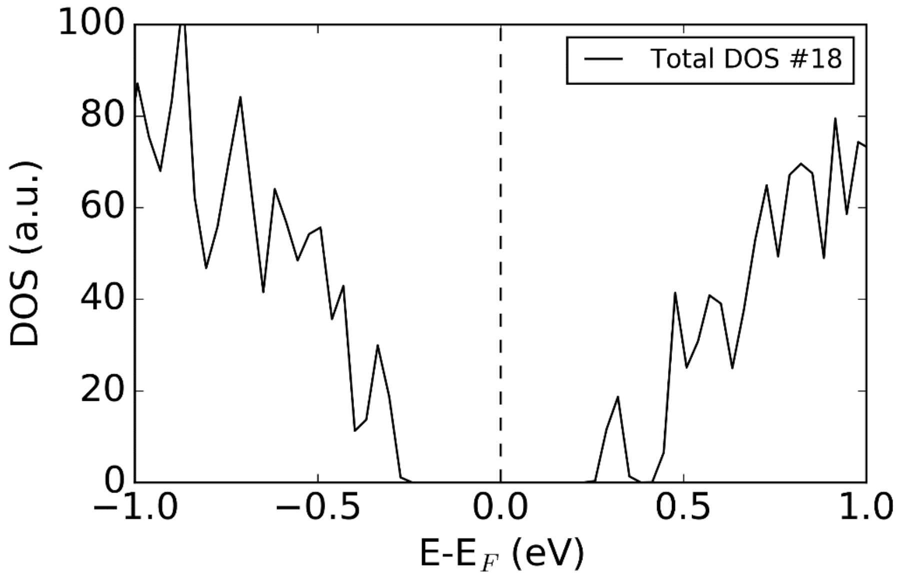

Supplementary Figure 21 | DOS glass model #18. Total electronic density of states near the top of the valence band and the bottom of the conduction band for the amorphous model #18. This glass structure has a shallow defect state located at 0.17 eV below the bottom of the conduction band, while the band gap of the model is 0.79 eV. The Fermi level lies approximately at the middle of the energy band gap.

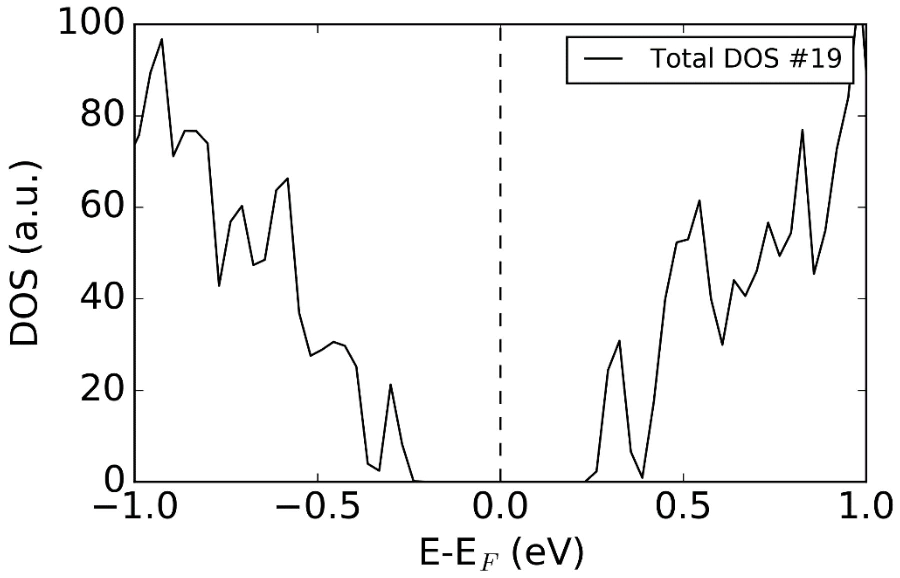

Supplementary Figure 22 | DOS glass model #19. Total electronic density of states near the top of the valence band and the bottom of the conduction band for the amorphous model #19. The calculated HOMO-LUMO band gap is 0.60 eV and this glass structure does not have a mid-gap defect state in the band gap. The Fermi level lies approximately at the middle of the energy band gap.

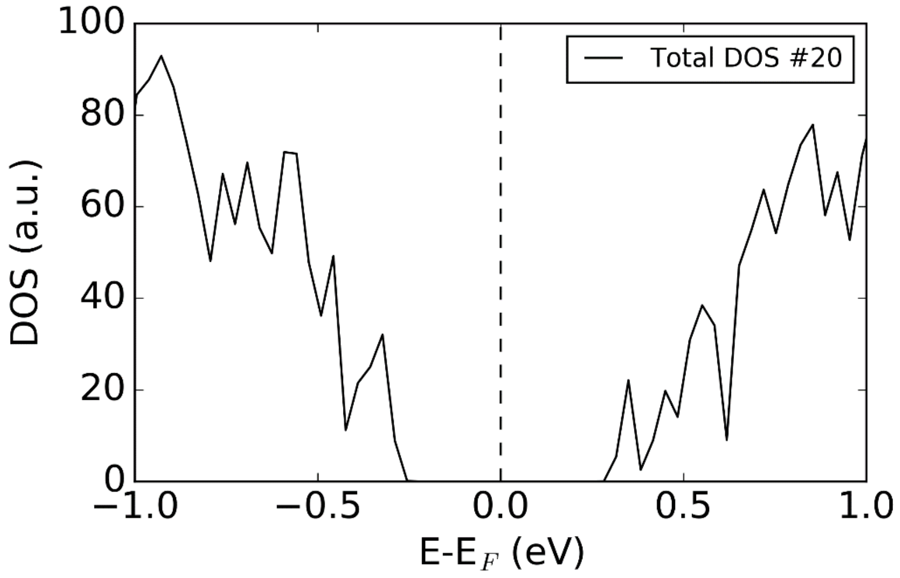

Supplementary Figure 23 | DOS glass model #20. Total electronic density of states near the top of the valence band and the bottom of the conduction band for the amorphous model #20. The calculated HOMO-LUMO band gap is 0.66 eV and this glass structure does not have a mid-gap defect state in the band gap. The Fermi level lies approximately at the middle of the energy band gap.

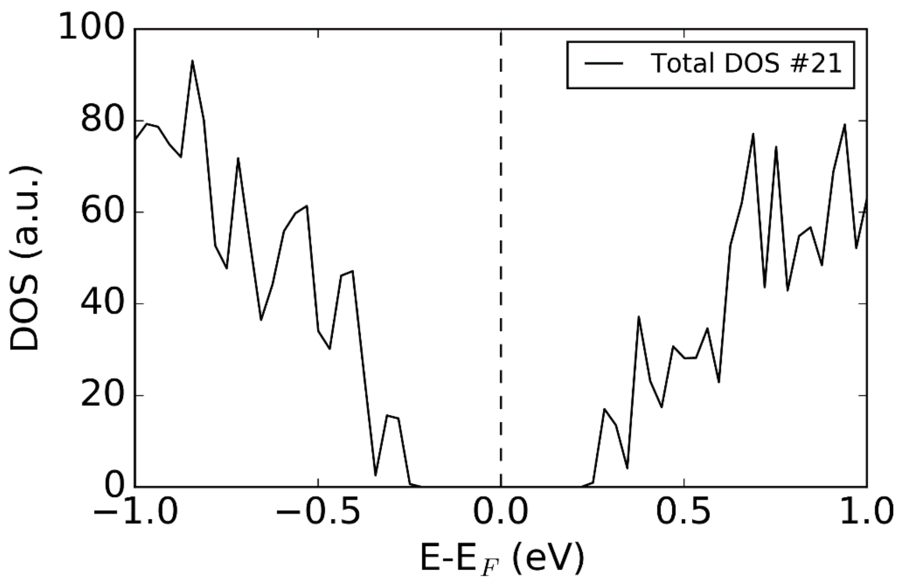

Supplementary Figure 24 | DOS glass model #21. Total electronic density of states near the top of the valence band and the bottom of the conduction band for the amorphous model #21. The calculated HOMO-LUMO band gap is 0.59 eV and this glass structure does not have a mid-gap defect state in the band gap. The Fermi level lies approximately at the middle of the energy band gap.

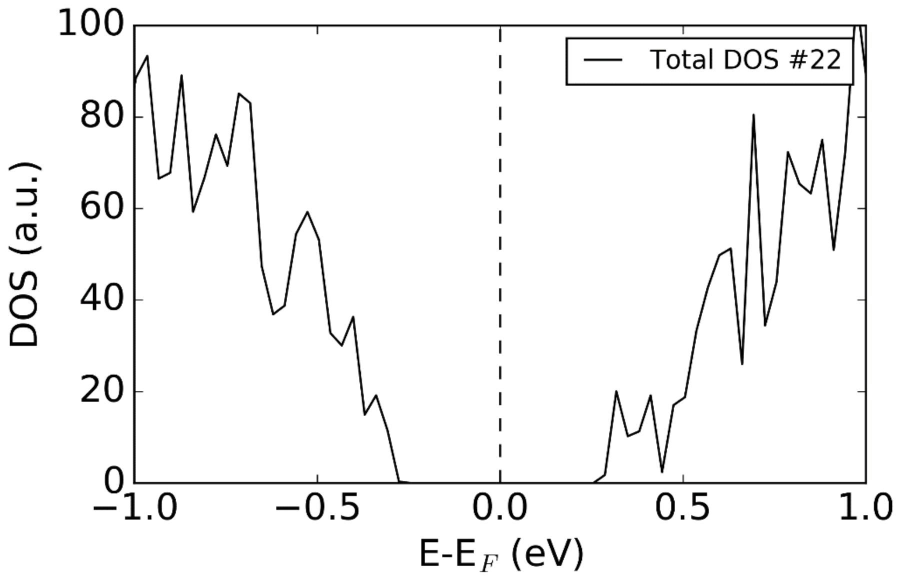

Supplementary Figure 25 | DOS glass model #22. Total electronic density of states near the top of the valence band and the bottom of the conduction band for the amorphous model #22. The calculated HOMO-LUMO band gap is 0.66 eV and this glass structure does not have a mid-gap defect state in the band gap. The Fermi level lies approximately at the middle of the energy band gap.

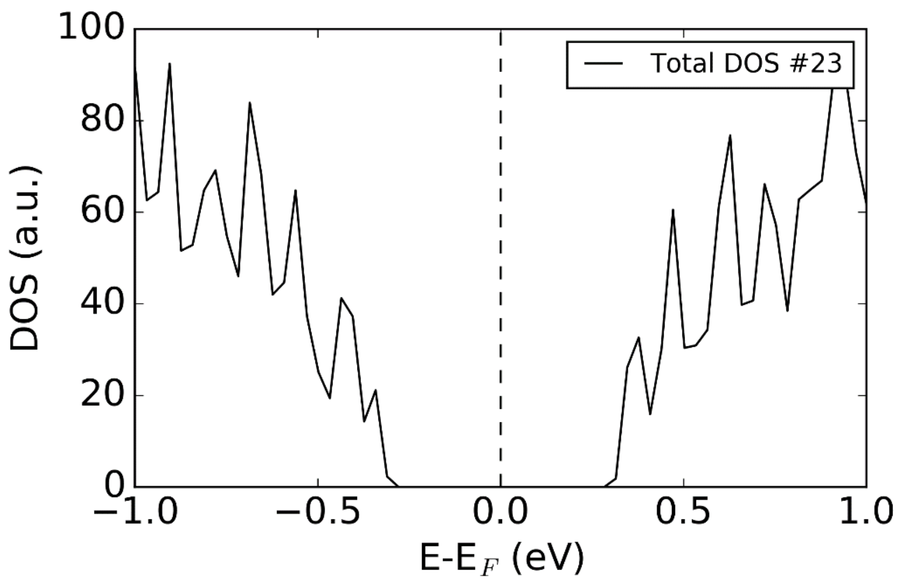

Supplementary Figure 26 | DOS glass model #23. Total electronic density of states near the top of the valence band and the bottom of the conduction band for the amorphous model #23. The calculated HOMO-LUMO band gap is 0.70 eV and this glass structure does not have a mid-gap defect state in the band gap. The Fermi level lies approximately at the middle of the energy band gap.

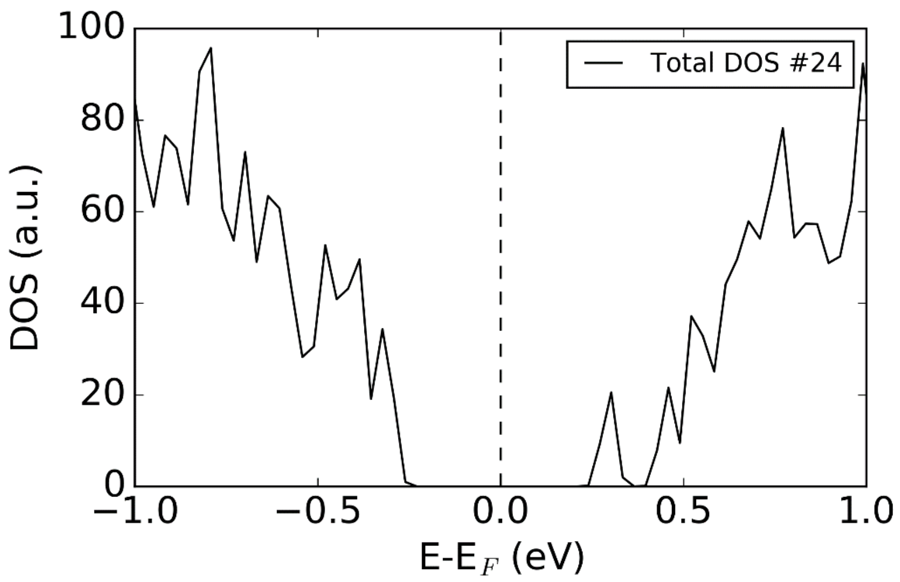

Supplementary Figure 27 | DOS glass model #24. Total electronic density of states near the top of the valence band and the bottom of the conduction band for the amorphous model #24. This glass structure has a shallow defect state located at 0.16 eV below the bottom of the conduction band, while the band gap of the model is 0.76 eV. The Fermi level lies approximately at the middle of the energy band gap.

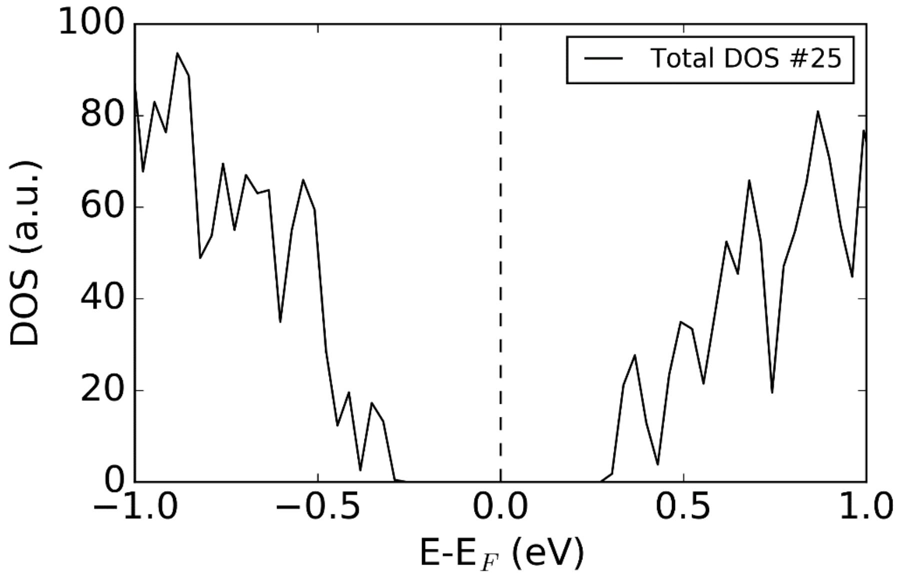

Supplementary Figure 28 | DOS glass model #25. Total electronic density of states near the top of the valence band and the bottom of the conduction band for the amorphous model #25. The calculated HOMO-LUMO band gap is 0.68 eV and this glass structure does not have a mid-gap defect state in the band gap. The Fermi level lies approximately at the middle of the energy band gap.

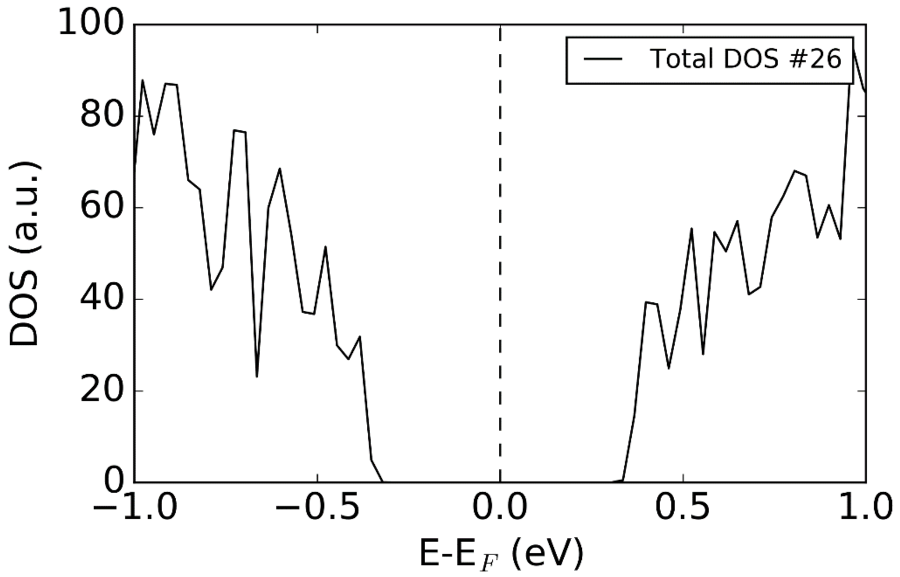

Supplementary Figure 29 | DOS glass model #26. Total electronic density of states near the top of the valence band and the bottom of the conduction band for the amorphous model #26. The calculated HOMO-LUMO band gap is 0.77 eV and this glass structure does not have a mid-gap defect state in the band gap. The Fermi level lies approximately at the middle of the energy band gap.

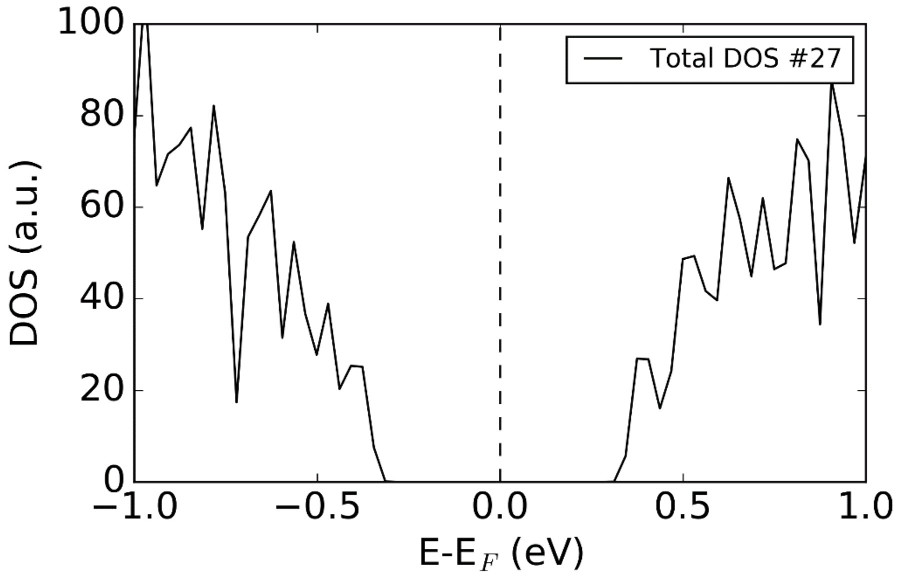

Supplementary Figure 30 | DOS glass model #27. Total electronic density of states near the top of the valence band and the bottom of the conduction band for the amorphous model #27. The calculated HOMO-LUMO band gap is 0.74 eV and this glass structure does not have a mid-gap defect state in the band gap. The Fermi level lies approximately at the middle of the energy band gap.

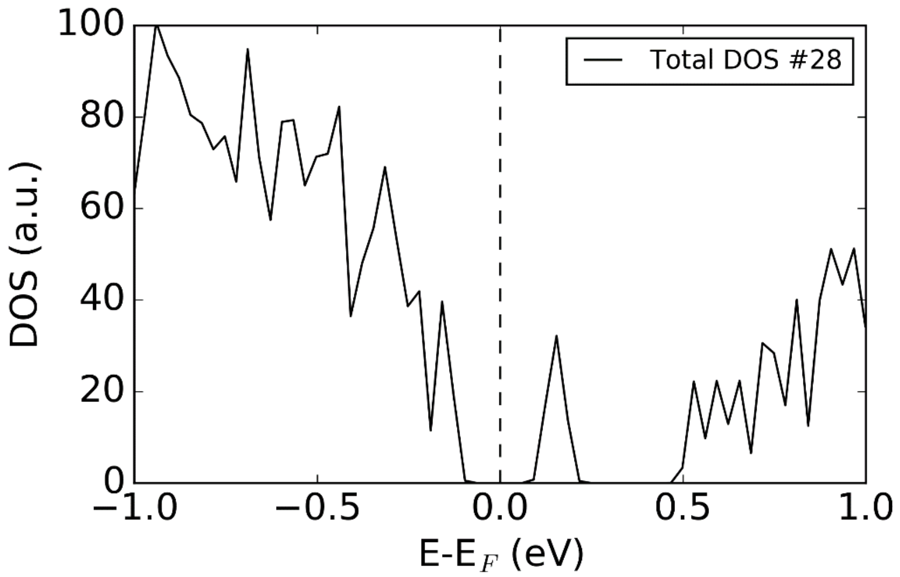

Supplementary Figure 31 | DOS glass model #28. Total electronic density of states near the top of the valence band and the bottom of the conduction band for the amorphous model #28. This glass structure has a mid-gap defect state located at 0.39 eV below the bottom of the conduction band, while the band gap of the model is 0.68 eV. The Fermi level lies deep in the band gap of the glass model, at a position between the mid-gap electronic state and the valence-band maximum.

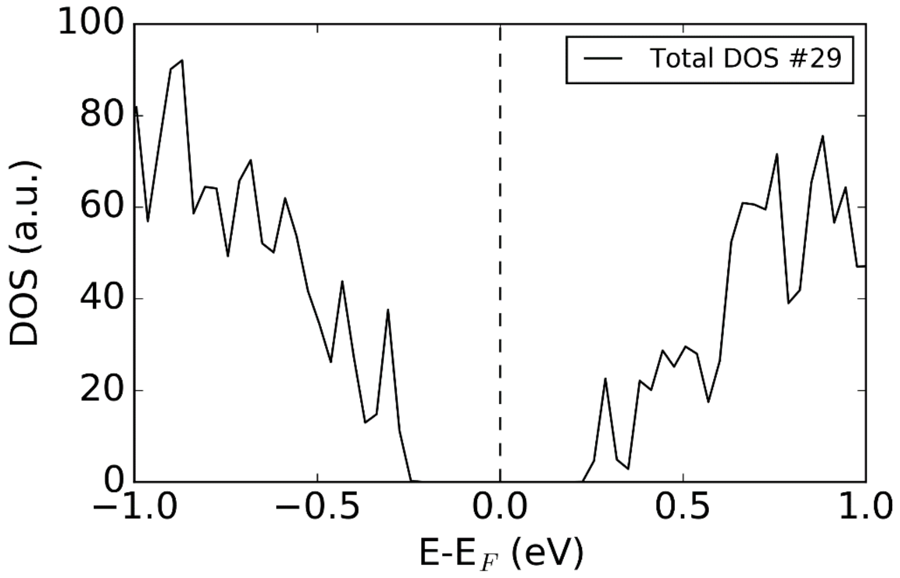

Supplementary Figure 32 | DOS glass model #29. Total electronic density of states near the top of the valence band and the bottom of the conduction band for the amorphous model #29. The calculated HOMO-LUMO band gap is 0.59 eV and this glass structure does not have a mid-gap defect state in the band gap. The Fermi level lies approximately at the middle of the energy band gap.

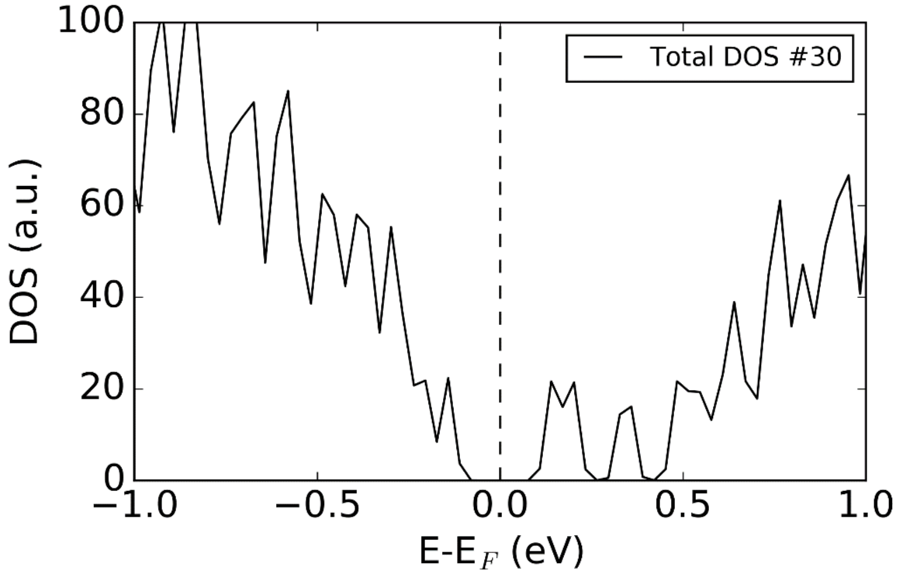

Supplementary Figure 33 | DOS glass model #30. Total electronic density of states near the top of the valence band and the bottom of the conduction band for the amorphous model #30. This glass structure has a mid-gap defect state located at 0.34 eV below the bottom of the conduction band, while the band gap of the model is 0.63 eV. Two more defect states, at 0.15 eV and 0.29 eV, also exist in the band gap of the glass structure. The Fermi level lies deep in the band gap of the glass model, at a position between the lowest mid-gap electronic state and the valence-band maximum.

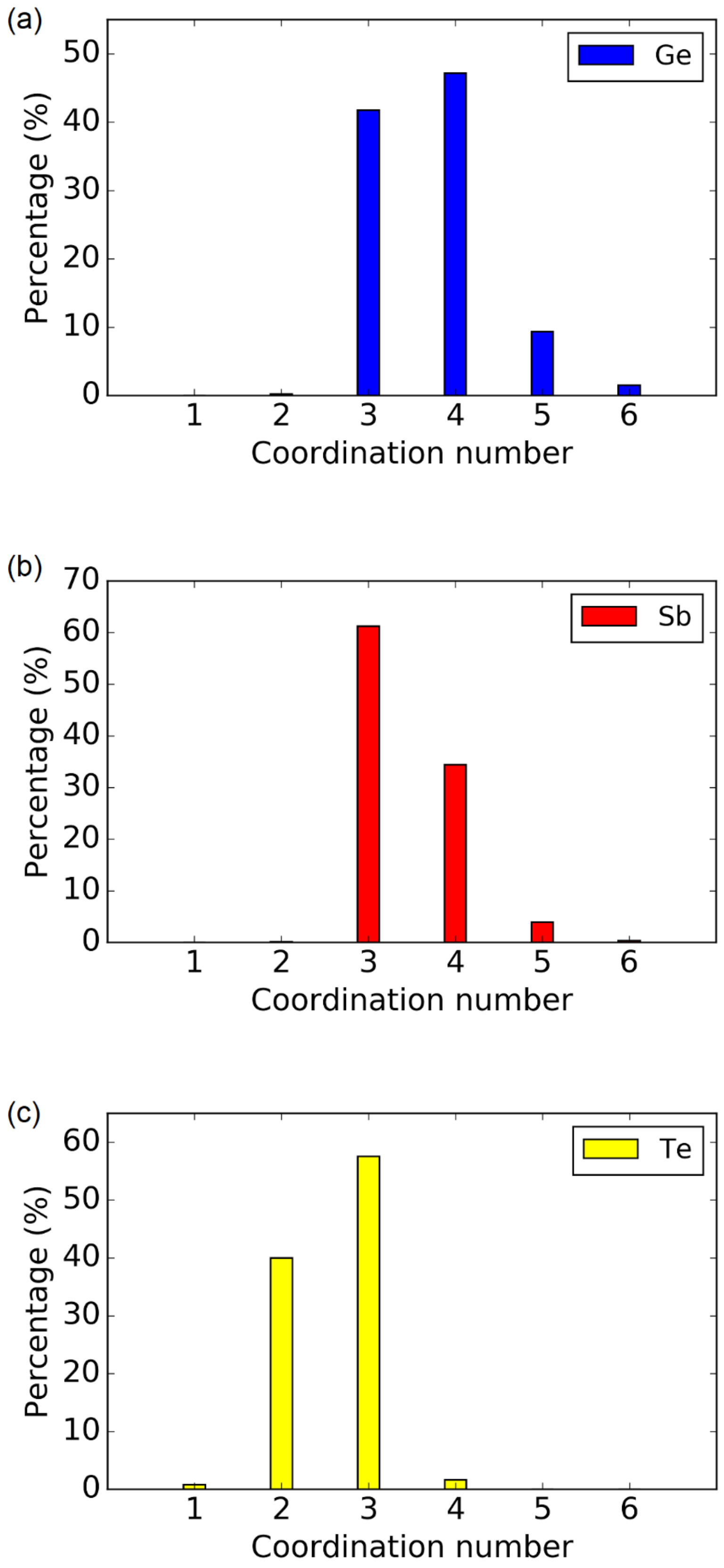

Supplementary Figure 34 | ELF analysis of local environments. Coordination-number histograms obtained from an Electron-Localization Function (ELF) analysis for the local environments of: **(a)** Ge atoms, **(b)** Sb atoms, and **(c)** Te atoms, averaged over the thirty 225GST glass models.

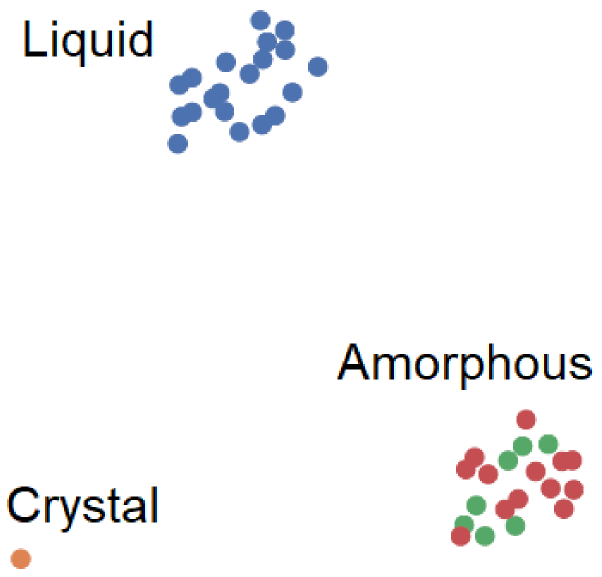

Supplementary Figure 35 | SOAP map of the glass models. Two-dimensional configurational SOAP map of liquid (blue points) and amorphous structures, with mid-gap electronic states (green points) and without defect states (red points) in their band gap, for the 225GST models generated in this study. The SOAP descriptor for an ideal 225GST crystalline structure (orange point) has been added to the map for the sake of comparison. Distances between two points on the map indicate the degree of structural similarity between the various structures; the closer, the more similar.

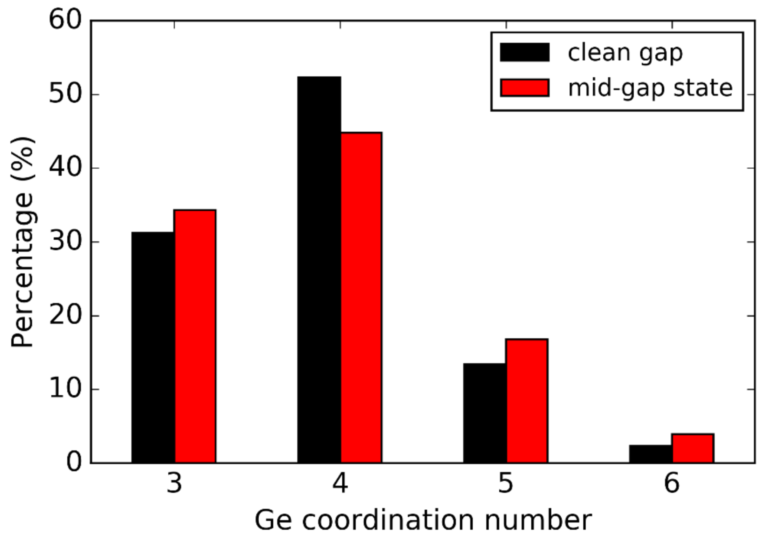

Supplementary Figure 36 | Differences in Ge local atomic geometries. Comparison of the local environments of Ge atoms with 3, 4, 5 and 6 other atoms in the nearest-neighbour positions between the glass structures without in-gap electronic states (clean band gap) and the glass structures with mid-gap defect states in the band gap. Glass models with mid-gap states have more defective Ge environments (3-, 5- and 6-coordinated Ge atoms) within their amorphous network. It should be noted that the coordination numbers around the Ge atoms were calculated by using a geometrical bond-distance cut-off of 3.2 Å.

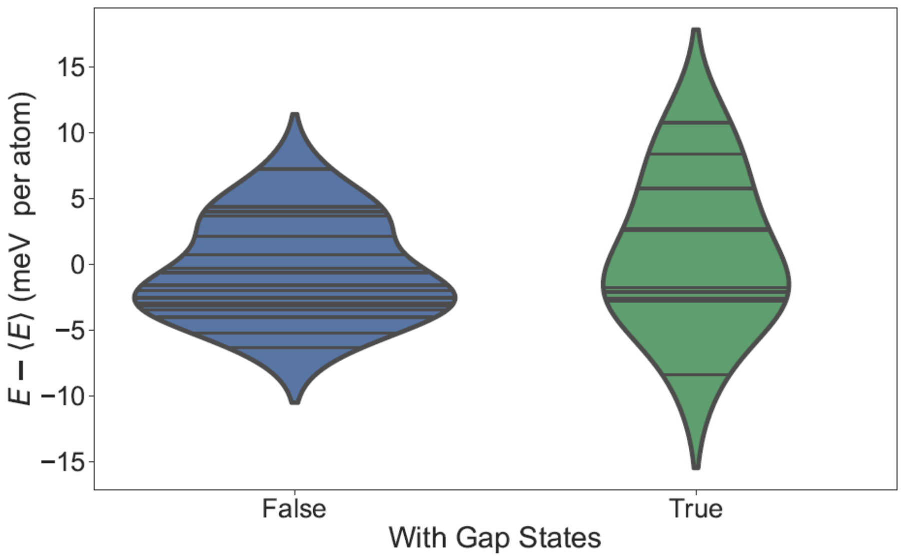

Supplementary Figure 37 | Total energy and in-gap electronic states. Comparison of the energetic character between the glass models with in-gap states (True) and without gap states (False) in the band gap. One can observe that the glass structures with in-gap states tend to be more unfavourable from an energetic perspective.

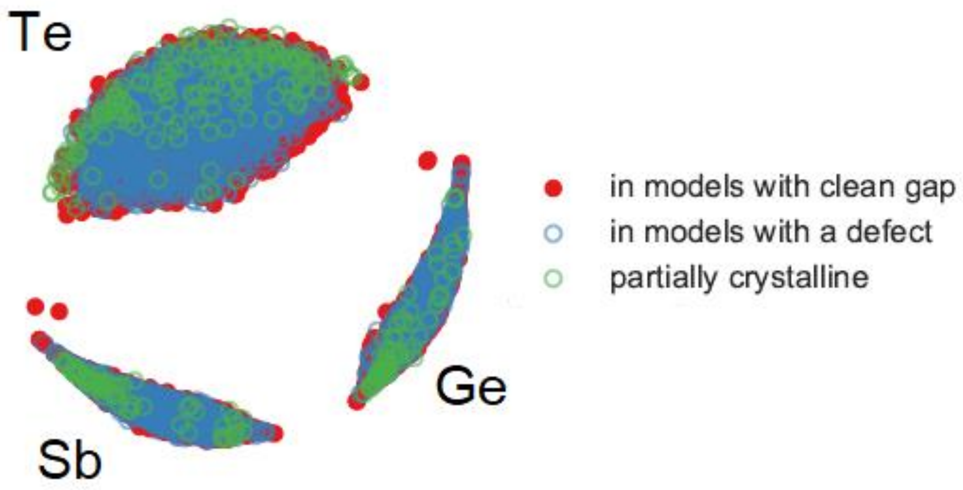

Supplementary Figure 38 | Coordination numbers of crystalline envrionments. Configurational SOAP map of the Ge, Sb, and Te local environments for each atomic species in the amorphous 225GST structures generated in this work, coloured by the nature of the structure in which the environment is found; partially crystalline (green empty circles), amorphous with mid-gap defect state (blue empty circles) and amorphous without in-gap electronic states (red circles).

Supplementary Tables

Supplementary Table 1 | Electronic-structure calculations. Calculated band gaps for the relaxed ground state of the 30 glass models generated in this work. The band gaps range from 0.55 to 0.79 eV. The calculated values agree very well with the experimentally reported values for amorphous 225GST[28,29], ranging between 0.6 and 0.8 eV, as well as with previous modelling studies[3,30]. 11 models have in-gap electronic states in their band gap, while the remaining 19 models have a kind of clean band gap. The position of the mid-gap defect is located, on average, at 0.34 eV below the bottom of the conduction band. The injection of an extra electron results in the formation of an electron centre, and the position of this state ranges from 0.33 to 0.61 eV, representing a deep electron trap. The energies for the position of mid-gap states, and corresponding electron traps, are measured from the bottom of the conduction band.

Glass model	Band gap (eV)	Mid-gap state	Position (eV)	Electron trap (eV)
1	0.72	NO	-	-
2	0.59	YES	0.27	0.49
3	0.64	NO	-	-
4	0.63	YES	0.30	0.54
5	0.62	YES	0.37	0.54
6	0.71	NO	-	-
7	0.61	NO	-	-
8	0.76	YES	0.21,0.35,0.45	0.41
9	0.78	YES	0.15,0.35	0.52
10	0.61	NO	-	-
11	0.55	NO	-	-
12	0.75	NO	-	-
13	0.62	YES	0.33	0.56
14	0.68	YES	0.27	0.61
15	0.61	NO	-	-
16	0.55	NO	-	-
17	0.60	NO	-	-
18	0.79	YES	0.17	0.67
19	0.60	NO	-	-
20	0.66	NO	-	-
21	0.59	NO	-	-
22	0.66	NO	-	-
23	0.70	NO	-	-

24	0.76	YES	0.16	0.69
25	0.68	NO	-	-
26	0.77	NO	-	-
27	0.74	NO	-	-
28	0.68	YES	0.39	0.33
29	0.59	NO	-	-
30	0.63	YES	0.15,0.29,0.34	0.37

Supplementary Table 2 | Total energy of the glass models. Total energy of the relaxed ground state for the 30 glass models generated in this work. The energy of the relaxed 225GST glasses with in-gap electronic states in the band gap is, on average, higher than that of the amorphous systems with no additional electronic states in their band gap.

Glass model	In-gap states	Total energy (hartree)
1	NO	-2086.605
2	YES	-2086.523
3	NO	-2086.682
4	YES	-2086.616
5	YES	-2086.672
6	NO	-2086.694
7	NO	-2086.683
8	YES	-2086.581
9	YES	-2086.680
10	NO	-2086.651
11	NO	-2086.639
12	NO	-2086.708
13	YES	-2086.551
14	YES	-2086.680
15	NO	-2086.684
16	NO	-2086.623
17	NO	-2086.601
18	YES	-2086.618
19	NO	-2086.564
20	NO	-2086.677

21	NO	-2086.602
22	NO	-2086.671
23	NO	-2086.597
24	YES	-2086.745
25	NO	-2086.666
26	NO	-2086.688
27	NO	-2086.655
28	YES	-2086.668
29	NO	-2086.721
30	YES	-2086.678

Supplementary References

1. Drabold, D. A. Topics in the theory of amorphous materials. *Eur. Phys. J. B* **68**, 1 (2009).
2. Akola J. & Jones, R. O. Structural phase transitions on the nanoscale: The crucial pattern in the phase-change materials $Ge_2Sb_2Te_5$ and GeTe. *Phys. Rev. B* **76**, 235201 (2007).
3. Caravati, S., Bernasconi, M., Kühne, T. D., Krack, M. & Parrinello, M. First-principles study of crystalline and amorphous $Ge_2Sb_2Te_5$ and the effects of stoichiometric defects. *J. Phys.: Condens. Matter* **21**, 255501 (2009).
4. Lee, T. H. & Elliott, S. R. The relation between chemical bonding and ultrafast crystal growth. *Adv. Mater.* **29**, 1700814 (2017).
5. Mocanu, F. C., Konstantinou, K., Lee, T. H., Bernstein, N., Deringer, V. L., Csányi, G. & Elliott, S. R. Modeling the phase-change memory material, $Ge_2Sb_2Te_5$, with a machine-learned interatomic potential. *J. Phys. Chem. B* **122**, 8998 (2018).
6. Kohara, S., Kato, K., Kimura, S., Tanaka, H., Usuki, T., Suzuya, K., Tanaka, H., Moritomo, Y., Matsunaga, T., Yamada, N., Tanaka, Y., Suematsu, H. & Takata, M. Structural basis for the fast phase change of $Ge_2Sb_2Te_5$: Ring statistics analogy between the crystal and amorphous states. *Appl. Phys. Lett.* **89**, 201910 (2006).
7. Bartók, A. P., Kondor R. & Csányi, G. On representing chemical environments. *Phys. Rev. B* **87**, 184115 (2013).
8. De, S., Bartók, A. P., Csányi, G. & Ceriotti, M. Comparing molecules and solids across structural and alchemical space. *Phys. Chem. Chem. Phys.* **18**, 13754 (2016).

9. Mavračić, J., Mocanu, F. C., Deringer, V. L., Csányi G. & Elliott, S. R. Similarity between amorphous and crystalline phases: The case of TiO_2. *J. Phys. Chem. Lett.* **9**, 2985 (2018).
10. Borg, I. & Groenen, P. J. F. Modern Multidimensional Scaling: Theory and Applications. Springer-Verlag: New York (2005).
11. Savin, A., Becke, A. D., Flad, J., Nesper, R., Preuss, H. & von Schnering, H. G. A new look at electron localization. *Angew. Chem., Int. Ed.* **30**, 409 (1991).
12. Silvi, B. & Savin, A. Classification of chemical bonds based on topological analysis of electron localization functions. *Nature* **371**, 683 (1994).
13. Kalikka, J., Akola, J., Jones, R. O., Kohara, S. & Usuki, T. Amorphous $Ge_{15}Te_{85}$: density functional, high-energy x-ray and neutron diffraction study. *J. Phys.: Condens. Matter* **24**, 015802 (2012).
14. Deringer, V. L., Zhang, W., Lumeij, M., Maintz, S., Wuttig, M., Mazzarello, R. & Dronskowski, R. Bonding nature of local structural motifs in amorphous GeTe. *Angew. Chem., Int. Ed.* **53**, 10817 (2014).
15. Xu, M., Cheng, Y. Q., Sheng, H. W. & Ma, E. Nature of atomic bonding and atomic structure in the phase-change $Ge_2Sb_2Te_5$ glass. *Phys. Rev. Lett.* **103**, 195502 (2009).
16. Mukhopadhyay, S., Sun, J., Subedi, A., Siegrist, T. & Singh, D. J. Competing covalent and ionic bonding in Ge-Sb-Te phase change materials. *Sci. Rep.* **6**, 25981 (2016).
17. Bouzid, A., Ori, G., Boero, M., Lampin, E. & Massobrio, C. Atomic-scale structure of the glassy $Ge_2Sb_2Te_5$ phase change material: A quantitative assessment via first-principles molecular dynamics. *Phys. Rev. B* **96**, 224204 (2017).
18. Kim, K. Y., Cho, D. Y., Cheong, B. K., Kim, D., Horii, H. & Han, S. Competing local orders in liquid and amorphous structures of $Ge_2Sb_2Te_5$: Influence of exchange-correlation functional. *J. Appl. Phys.* **113**, 134302 (2013).
19. Bartók, A. P., Payne, M. C., Kondor, R. & Csányi, G. Gaussian approximation potentials: The accuracy of quantum mechanics, without the electrons. *Phys. Rev. Lett.* **104**, 136403 (2010).
20. Bartók, A. P. & Csányi, G. Gaussian approximation potentials: A brief tutorial introduction. *Int. J. Quant. Chem.* **115**, 1051 (2015).
21. Njoroge, W. K., Wöltgens, H. W. & Wuttig, M. Density changes upon crystallization of $Ge_2Sb_{2.04}Te_{4.74}$ films. *J. Vac. Sci. Technol. A* **20**, 230 (2002).
22. Konstantinou, K., Sushko, P. V. & Duffy, D. M. Modelling the local atomic structure of molybdenum in nuclear waste glasses with *ab initio* molecular dynamics simulations. *Phys. Chem. Chem. Phys.* **18**, 26125 (2016).

23. Guidon, M., Hutter, J. & VandeVondele, J. Robust periodic Hartree-Fock exchange for large-scale simulations using Gaussian basis sets. *J. Chem. Theory Comput.* **5**, 3010 (2009).
24. VandeVondele, J., Krack, M., Mohamed, F., Parrinello, M., Chassaing, T. & Hutter, J. QUICKSTEP: Fast and accurate density functional calculations using a mixed Gaussian and plane waves approach. *Comput. Phys. Commun.* **167**, 103 (2005).
25. Goedecker, S., Teter, M. & Hutter, J. Separable dual-space Gaussian pseudopotentials. *Phys. Rev. B* **54**, 1703 (1996).
26. Guidon, M., Hutter, J. & VandeVondele, J. Auxiliary density matrix methods for Hartree-Fock exchange calculations. *J. Chem. Theory Comput.* **6**, 2348 (2010).
27. Konstantinou, K., Duffy, D. M. & Shluger, A. L. Structure and luminescence of intrinsic localized states in sodium silicate glasses. *Phys. Rev. B* **94**, 174202 (2016).
28. Lee, B. S., Abelson, J. R., Bishop, S. G., Kang, D. H., Cheong, B. K. & Kim, K. B. Investigation of the optical and electronic properties of $Ge_2Sb_2Te_5$ phase change material in its amorphous, cubic, and hexagonal phases. *J. Appl. Phys.* **97**, 093509 (2005).
29. Kato, T. & Tanaka, K. Electronic properties of amorphous and crystalline $Ge_2Sb_2Te_5$ films. *Jpn. J. Appl. Phys.* **44**, 7340 (2005).
30. Kozyukhin, S. A., Nguyena, H. P., Tsendin, K. D. & Prikhodko, O. Y. Optical properties of amorphous thin films Ge–Sb–Te phase-change materials. *Int. J. Adv. Res. in Phys. Sci.* **3**, 1 (2016).
